# Post-thaw dimethyl sulfoxide reduction in autologous peripheral blood progenitor cell suspensions

**DOI:** 10.1016/j.htct.2025.103965

**Published:** 2025-08-25

**Authors:** Miroslava Jandová, Pavel Měřička, Jiří Gregor, Miriam Lánská, Aleš Bezrouk, Dana Čížková, Jakub Radocha

**Affiliations:** a University Hospital Hradec Králové, Tissue Bank, Czechia; b Charles University, Faculty of Medicine in Hradec Králové, Department of Histology and Embryology, Czechia; c University Hospital Hradec Králové, 4th Department of Internal Medicine - Hematology Czech Republic, Czechia; d Charles University, Faculty of Medicine in Hradec Králové, Department of Medical Biophysics, Czechia

**Keywords:** Peripheral blood progenitor cells, Cryopreservation, DMSO reduction, Amyloidosis, Cell recovery

## Abstract

**Background and objectives:**

Dimethyl sulfoxide has become the most common cryoprotectant used for cryopreservation of hematopoietic progenitor cells because of its efficiency, regardless of its potentially toxic side effects. Its application is considered safe, provided that the daily dose administered does not exceed 1 gram per kilogram of patient weight. Indications for its reduction after thawing are limited to patients with high risk of malignant arrhythmia and those with severely impaired renal function. However, dimethyl sulfoxide reduction can lead to the loss of viable progenitors.

**Methods:**

A retrospective study of viable hematopoietic progenitor cell recovery after dimethyl sulfoxide reduction was performed with 13 patients (nine men, four women) with secondary amyloidosis in multiple myeloma (*n* = 9), primary amyloid light chain amyloidosis (*n* = 3), or severe adverse reaction at the beginning of the hematopoietic progenitor cell concentrate infusion (*n* = 1). The Wilcoxon signed-rank test was used.

**Results:**

The results of the dimethyl sulfoxide reduction process showed a high recovery of viable nucleated cells (median: 120.85 %), and of viable mononuclear cells (median: 104.53 %). There was a significant decrease in total number of viable CD34^+^ cells in comparison with data obtained after original collection (median: 51.49 %). No significant decrease in colony-forming unit capacity was observed after dimethyl sulfoxide reduction (median: 93.37 %).

**Conclusion:**

The dimethyl sulfoxide removal process and total process recoveries revealed considerable individual variability. To minimize the risk of prolonged engraftment or non-engraftment, it is important to apply this process only to high-risk patients.

## Introduction

After a period of using glycerol for the cryopreservation of hematopoietic progenitor cells (HPCs) [1], dimethyl sulfoxide (DMSO) has become the most commonly used cryoprotectant for this purpose because of its high cryoprotective efficiency and rapid penetration across cell membranes [2, 3, 4]. The great advantage of its use is the possibility of infusing the thawed cell product without removing the cryoprotectant, which was not possible with glycerol. However, the potential toxicity of DMSO has given rise to debates on the safety of its use. Cases of adverse reactions such as increased heart or respiratory rate, facial flushing, increased or decreased blood pressure, dyspnea, nausea, and vomiting have been described after infusion of HPC concentrates containing DMSO [3, 4, 5, 6, 7, 8, 9]. DMSO toxicity is dose-dependent. Therefore, strategies are being developed to neutralize the toxicity, to reduce the concentration of the cryoprotectant, or to wash it off before clinical administration. Infusion of an HPC product containing DMSO is generally accepted as safe in concentrations below 10 % (v/v) [10] under the condition that the maximal daily dose of DMSO does not exceed 1 g per kg patient body weight [11,12]. While the above-mentioned symptoms usually cause only transient discomfort for the patient, serious adverse reactions have been described in patients with preexisting cardiovascular, respiratory, renal or central nervous system diseases, sometimes with fatal outcomes [6, 7, 8,13]. In the practice of University Hospital Hradec Králové, the most frequent medical indications for DMSO reduction are chronic renal failure caused by secondary amyloidosis in multiple myeloma and primary or secondary amyloidosis of the heart.

The most widely used technique in DMSO washing is gradual dilution of the cell suspension with its subsequent centrifugation and the addition of cryoprotectant-free solution [14,15]. The degree of dilution and the composition of the washing solution are usually adjusted to minimize osmotic changes. The components of the washing medium should be acceptable from a clinical point of view, i.e., it should not contain components of animal origin and should contain registered drugs, and use CE-certified medical devices, or products approved by the national competent authority (State Institute for Drug Control in the Czech Republic) [15]. In clinical practice, certified saline solutions/electrolytes, such as 0.9 % NaCl, Normosol-R® (Hospira, Inc., USA), Plasma-Lyte 148® (Baxter, USA), Ringer's solution (B. Braun, Germany) with dextran-40 (5–10 %), human serum albumin (1–5 %), hydroxyethyl starch (HES - 3–6 %), or acid citrate dextrose anticoagulant are acceptable. These media are often supplemented with dextran-40, HES, or human serum albumin at various concentrations [16, 17, 18, 19].

Commercially available closed automatic systems developed for hematopoietic cell grafts, which are usually characterized by large-transplanted volumes, can also be used to wash out cryoprotectants. Examples of such systems are devices based on the principle of dilution and subsequent centrifugation, e.g., the COBE® 2991 Cell Processor (Terumo BCT, Inc.), Sepax S-100® (Sepax 2 S-100), and Biosafe SA® (GE HealthCare) [13,15,17,19,20], or on the principle of dilution and subsequent filtration, e.g., the Haemonetics ACP215 Automated Cell Processor® (Haemonetics Corp), CytoMate® (Baxter/Nexell), or Lovo® (Fresenius Kabi) [13,15,21,22].

Our clinical center has long experience with autologous HPC transplantation in multiple myeloma [23], and the infused DMSO dose is far below the limit [24] in most cases. A controlled study performed by Horacek et al. [25] did not report any differences in monitored vital functions between infusions of autologous and allogeneic HPC concentrates. Nevertheless, in a minority of cases, it was necessary to split the HPC dose over several days [24,26].

This retrospective study reviewed cases of primary or secondary amyloidosis as a complication of multiple myeloma treated by HPC autologous transplantation requiring DMSO reduction. Data regarding the influence of the freezing/thawing and DMSO reduction processes on the content of nucleated cells (NC), mononuclear cells (MNC), and CD34^+^ cells were analyzed. This analysis included pre- and post-process viability, the recovery of viable cells, and repopulation potency, as measured by the colony-forming unit-granulocyte macrophage (CFU-GM) assay, for samples contained within one 100 mL cryobag.

## Methods and materials

### *Patients and study design*

**Figure 1:**
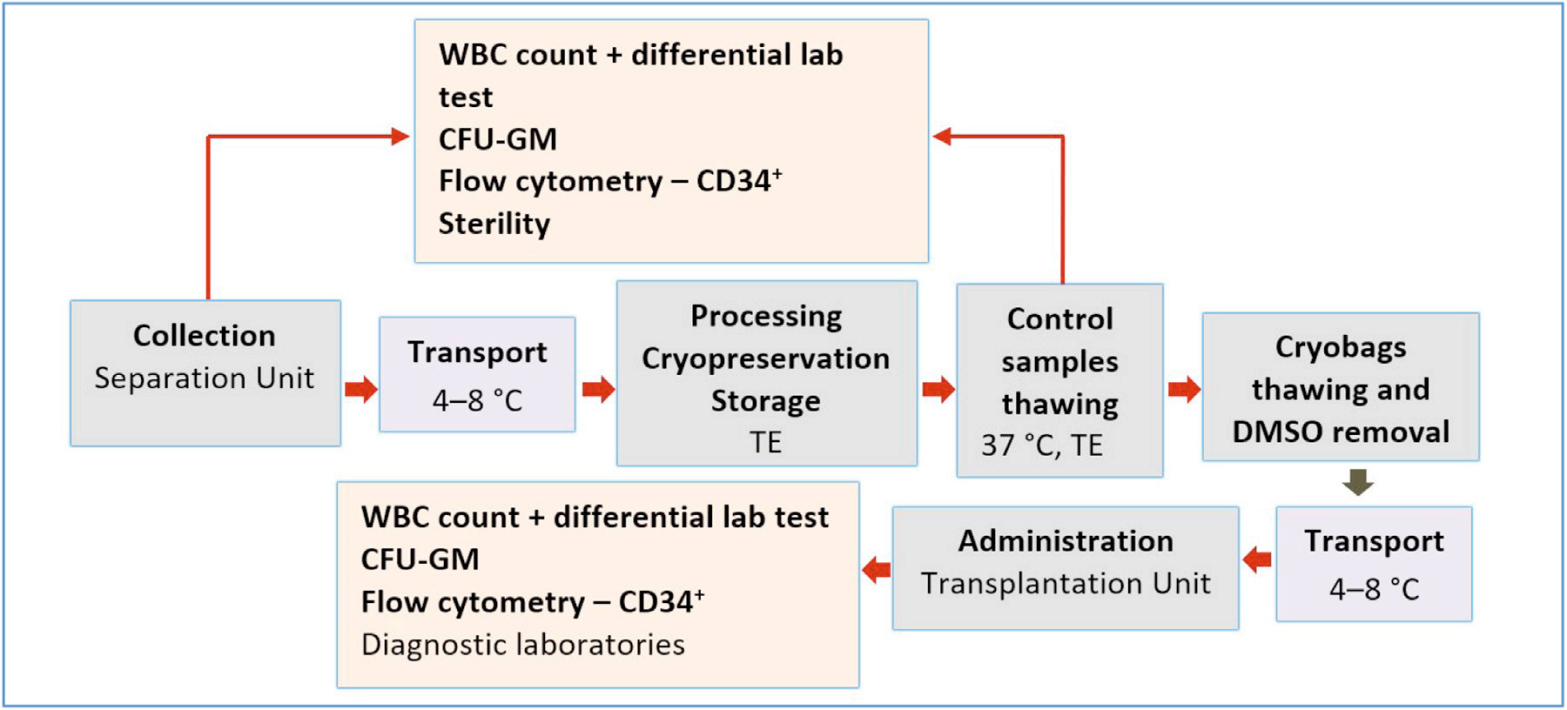
Diagram of hematopoietic progenitor cell collection, processing, and application.

WBC: white blood cell; CFU-GM: colony forming unit-granulocyte macrophage; DMSO: dimethyl sulfoxide

A retrospective study of the influence of freezing/thawing and the DMSO removal process on HPC concentrate parameters was conducted in 2013–2022. Thirteen patients were included. The inclusion criteria this study were complete documentation, initial NC concentration not exceeding 400 × 10^9^/L, and processing within 24 h after collection. The DMSO was reduced to approximately a quarter of the initial concentration and no more than two 100 mL bags were infused per day. For the purpose of this analysis, data from only one washing process of one 100 mL cryobag from the total of three or four cryobags of HPC concentrate obtained by one leukapheresis and stored for clinical application were compared. The process of HPC leukapheresis, transport, processing, and application is presented in Figure 1.

### *HPC stimulation and collection*

The HPCs were collected by leukapheresis after mobilization by cyclophosphamide (2.5 g per m^2^ of the patient body surface area) and granulocyte colony-stimulating factor (10 μg per kg of the patient weight) at a separator, namely Cobe Spectra or Spectra Optia (Terumo BCT, USA). Melphalan (140 or 200 mg per m^2^ of the patient body surface area) was used for conditioning.

### *HPC cryopreservation*

After transport to the Tissue Establishment, collected HPCs were processed in a laminar flow cabinet and under a laminary ceiling that adhered to Grade A purity with Class B background within 24 h of the harvest. A standard cryopreservation protocol for autologous HPCs using CE-certified DMSO (WAK Chemie GmbH, FRG) in a final concentration of 10 % (v/v), and HES (Voluven 10 %, Fresenius Kabi, GmbH, FRG) in the final concentration of 5 % supplemented with 20 % (v/v) human serum albumin (5 mL per 100 mL) were used. Three or four cryobags containing 70–100 mL of HPC suspension were created from the initial collection bag with a mean volume of 194 mL (154–205 mL) and were frozen in a programmable freezer (Planer Biomed, England) with a cooling rate of 1 °C/min to −90 °C and 5 °C/min to −150 °C. Cryopreserved HPCs were stored in the vapor phase of liquid nitrogen at a temperature below −160 °C in a biological container (Chart MVE, USA) with automatic filling and continuous temperature recording. The average time of storage was 103 days (range: 26–679 days).

### *DMSO reduction*

According to the results of estimation of the dose CD34^+^ cells from thawed control samples, the treating physician decided to use one or more bags. Metal cassettes containing bags with cryopreserved HPCs were removed from the storage container and thawed in a water bath at 37 °C. The thawing of each bag took approximately five minutes. The thawed bags were transferred to a clean room and further processed in the laminar flow cabinet (Grade A with Class B background). The total volume of each bag (mean 98 mL; range: 70–100 mL) was transferred to the washing bag, and was mixed with 258 mL of HES and 42 mL of ACD-A solution (Fresenius Kabi, GmbH, FRG). Then, the cell suspension was centrifuged for 20 min at 400 g at 4 °C in the adjacent room (Grade C). In all, 300 mL of the supernatant was removed again in the laminar flow cabinet after the centrifugation. The total time for the DMSO removal of each bag was approximately one hour. Bags containing washed HPCs were appropriately labeled and transferred to the clinical department in an insulated box at a temperature of 2–8 °C. The content was administered to the patient within two hours after thawing, without any complications. The infusion time of each bag was approximately ten minutes.

### *Sampling and quality control tests*

The following quality parameters of collected, cryopreserved, and DMSO‑depleted concentrates were determined: hematocrit and blood count including detailed white blood cell differential, total number of viable NC (TNC), MNC, and CD34^+^, CFU-GM, and sterility.

Hematological parameters were determined by an automated hematological analyzer Sysmex XN3000 (Sysmex, Japan). CD34^+^ phenotyping and viability determination was performed with the flow cytometer FACS (fluorescence-activated cell sorting) Navios (Beckman Coulter, USA) using SW Kaluza, Version 1.2 (Beckman Coulter, USA). Cell suspension was incubated with the anti-CD34-PE and anti-CD45-FITC monoclonal antibodies (Beckman Coulter, USA), and 7-AAD (Beckman Coulter, USA) as a vital dye.

Sterility testing was performed using a Bactalert (type BTA 3D 240, SW version B 50, BioMérieux, France) automatic microbial detection system situated in a clean room (Grade A with a Class B background) according to Czech Pharmacopoiea [27].

During CFU-GM assay in a biohazard safety cabinet certified for level II handling of biological materials, the defined amount of HPC suspension was diluted in Iscove's modified Dulbecco's medium (Sigma Aldrich, Czech Republic) and then cultured in a semi-solid matrix (Metho-Cult™, StemCell™ Technologies, USA) in Petri dishes. Cultivation took place in an incubator set at 37 °C with 5 % CO_2_ in air and ≥95 % humidity (BBD Herasafe, USA). An inverted microscope (Olympus CK 40, Japan) was used for colony counting. To perform counting of CFU-GM, colonies were observed after 14 days in culture, using 10× objective (50–100× magnification) according to Czech Pharmacopoiea [28].

### *Cell recovery calculation*

Values of individual parameters were compared: total number of viable NC, MNC, and CD34^+^ cells after collection, after cryopreservation, and after DMSO reduction. Recoveries of the individual phases of the DMSO reduction process such as: (1) freezing/thawing process, (2) DMSO removal, and (3) the total process (freezing/thawing + DMSO removal) were calculated according to the following equation:

$$Recovery\;=\;\frac{parameter\;x\;viability\;post-process}{parameter\;x\;viability\;pre-process}\;x\;100$$

### *Statistical methods*

The processes described above were evaluated using total numbers of viable NC, MNC, CD34^+^ cells and CFU-GM. The data were statistically evaluated using MS Excel 2016 (Microsoft Corp., Redmond, WA, USA) and NCSS 10 statistical software (2015, NCSS, LLC., Kaysville, UT, USA, and available online: ncss.com/software/ncss [accessed on 21 April 2023]). Because the measured data did not show a normal distribution, the median and the first and third quartiles (1st Q, 3rd Q) were utilized as descriptive statistics. Bonferroni correction of the alpha significance level was used for multiple data comparisons. The data were compared using the Wilcoxon signed-rank test at the corrected alpha value α = 0.017. Correlation analysis between the number of viable CD34^+^ cells and CFU-GM after thawing and DMSO removal was conducted.

## Results

Study inclusion criteria were met by 13 patients (nine men and four women) with an average age of 58 years (range: 44–70 years) and weight of 80 kg (range: 52–103 kg). The retrospective study period was 2013–2020. The whole process of HPC collection, transport, processing, and administration is presented in Figure 1. Twelve patients had a diagnosis of primary amyloidosis or secondary amyloidosis as a complication of multiple myeloma, and one patient (No. 3) had an allergic adverse reaction to DMSO at the beginning of HPC infusion (Table 1).

**Table 1:** Data of patients included in the retrospective study.

Patient number	Sex	Age (year)	Weight (kg)	Diagnosis/reason for washing
1	male	55	81	Multiple myeloma - secondary amyloidosis - suspected amyloidosis of heart
2	female	61	52	Primary amyloidosis of bone marrow, liver, and kidneys
3	male	48	94	Multiple myeloma - allergic adverse reaction (DMSO)
4	male	56	91	Multiple myeloma - secondary amyloidosis - suspected amyloidosis of heart
5	female	55	80	Multiple myeloma - secondary amyloidosis of kidneys
6	female	57	61	Multiple myeloma - secondary amyloidosis - suspected amyloidosis of heart
7	male	69	93	Primary amyloidosis of liver and lungs
8	female	51	58	Multiple myeloma - secondary amyloidosis of gastrointestinal tract and kidneys
9	male	44	102	Multiple myeloma - secondary amyloidosis - suspected amyloidosis of heart
10	male	58	84	Multiple myeloma - secondary amyloidosis of duodenum and heart
11	male	66	77	Multiple myeloma - secondary amyloidosis of gastrointestinal tract
12	male	59	67	Primary amyloidosis of bone marrow and kidneys
13	male	70	103	Multiple myeloma - secondary amyloidosis - suspected amyloidosis of heart

### *Cell parameters of key processes*

Descriptive statistics (median, 1st and 3rd quartile) of HPC key parameters characterizing individual processes, such as leukapheresis, freezing/thawing, and DMSO removal process are presented in Table 2, Table 3, Table 4. These parameters were compared using the Wilcoxon signed-rank test with the aim of finding any significant differences between the values characterizing the pre- and post-processes. The freezing/thawing process significantly reduced the NC and MNC viability, TNC as well as the CFU-GM dose per kg of patient weight. The doses of MNC per kg and CD34^+^ cells per kg were not affected, and the post-thaw MNC percentage increased significantly (Table 2).

**Table 2:** Descriptive statistics of the data characterizing freezing/thawing process, and their comparison using the Wilcoxon signed-rank test. Data obtained from 13 patients.

Parameter	Pre-process (leukapheresis) median (Q1; Q3)	Post-process (freezing-thawing) median (Q1; Q3)	p-value
TNC/kg (x 10^8^)	2.46 (1.96; 4.01)	1.79 (1.24; 3.04)	0.006*
NC viability ( %)	100 (100; 100)	82 (74; 95)	0.002*
TMNC/kg (x 10^8^)	1.51 (0.80; 1.78)	1.40 (1.01; 2.09)	0.364
MNC viability ( %)	100 (100; 100)	87 (92; 99)	0.002*
MNC from TNC ( %)	56 (33; 66)	70 (60; 83)	0.002*
CD34^+^/kg (x 10^6^)	3.71 (1.64; 6.15)	3.88 (1.42; 6.84)	0.529
CD34^+^ from TNC ( %)	1.33 (0.80; 2.00)	1.63 (0.87; 2.09)	0.014*
CFU-GM/kg (x 10^5^)	2.97 (1.71; 4.05)	1.68 (1.42; 2.91)	0.002*

⁎ Statistically significant difference TNC: total number of viable nucleated cells; NC: nucleated cells; TMNC: total number of viable mononuclear cells; MNC mononuclear cells; CFU-GM: colony forming unit-granulocyte macrophage.

**Table 3:** Descriptive statistics of the data characterizing dimethyl sulfoxide removal process, and their comparison using the Wilcoxon signed-rank test. Data obtained from 13 patients.

Parameter	Pre-process (freezing-thawing) median (Q1; Q3)	Post-process (DMSO removal) median (Q1; Q3)	P value
TNC/kg (x 10^8^)	1.79 (1.24; 3.04)	1.79 (1.37; 2.20)	0.576
NC viability ( %)	82 (74; 95)	78 (69; 85)	0.025
TMNC/kg (x 10^8^)	1.40 (1.01; 2.09)	1.40 (0.87; 1.75)	0.402
MNC viability ( %)	87 (92; 99)	87 (83; 92)	0.002*
MNC from TNC ( %)	70 (60; 83)	63 (47; 83)	0.081
CD34^+^/kg (x 10^6^)	3.88 (1.42; 6.84)	1.76 (1.13; 3.65)	0.018
CD34^+^ from TNC ( %)	1.63 (0.87; 2.09)	0.90 0.51; 1.51)	0.002*
CFU-GM/kg (x 10^5^)	1.68 (1.42; 2.91)	1.82 (1.31; 2.71)	0.133

⁎ Statistically significant difference TNC: total number of viable nucleated cells; NC: nucleated cells; TMNC: total number of viable mononuclear cells; MNC mononuclear cells; CFU-GM: colony forming unit-granulocyte macrophage.

**Table 4:** Descriptive statistics of the data characterizing total dimethyl sulfoxide removal process, and their comparison using Wilcoxon signed-rank test. Data obtained from 13 patients.

Parameter	Pre-process (leukapheresis) median (Q1; Q3)	Post-process (DMSO removal) median (Q1; Q3)	P value
TNC/kg (10^8^)	2.46 (1.96; 4.01)	1.79 (1.37; 2.20)	0.036
NC viability (%)	100 (100; 100)	78 (69; 85)	0.002*
TMNC/kg (10^8^)	1.51 (0.80; 1.78)	1.40 (0.87; 1.75)	0.944
MNC viability (%)	100 (100; 100)	87 (83; 92)	0.002*
MNC from TNC (%)	56 (33; 66)	63 (47; 83)	0.036
CD34^+^/kg (10^6^)	3.71 (1.64; 6.15)	1.76 (1.13; 3.65)	0.010*
CD34^+^ from TNC (%)	1.33 (0.80; 2.00)	0.90 0.51; 1.51)	0.003*
CFU-GM/kg (10^5^)	2.97 (1.71; 4.05)	1.82 (1.31; 2.71)	0.006*

⁎ Statistically significant difference TNC: total number of viable nucleated cells; NC: nucleated cells; TMNC: total number of viable mononuclear cells; MNC mononuclear cells; CFU-GM: colony forming unit-granulocyte macrophage.

The DMSO removal process significantly reduced MNC viability and the percentage of CD34^+^ cells from leukocytes. Other parameters were not significantly affected (Table 3).

Data from leukapheresis indicated that the DMSO removal process significantly reduced the number of viable NC and MNC, the percentage of CD34^+^ cells from leukocytes, CD34^+^ cells, and CFU-GM dose per patient body weight (Table 4).

### *Results of sterility determination*

Sterility was verified in all samples after cryopreservation and after DMSO removal. All evaluated samples were sterile.

### *Post-thaw recovery of key hematopoietic progenitor cells parameters*

**Figure 2:**
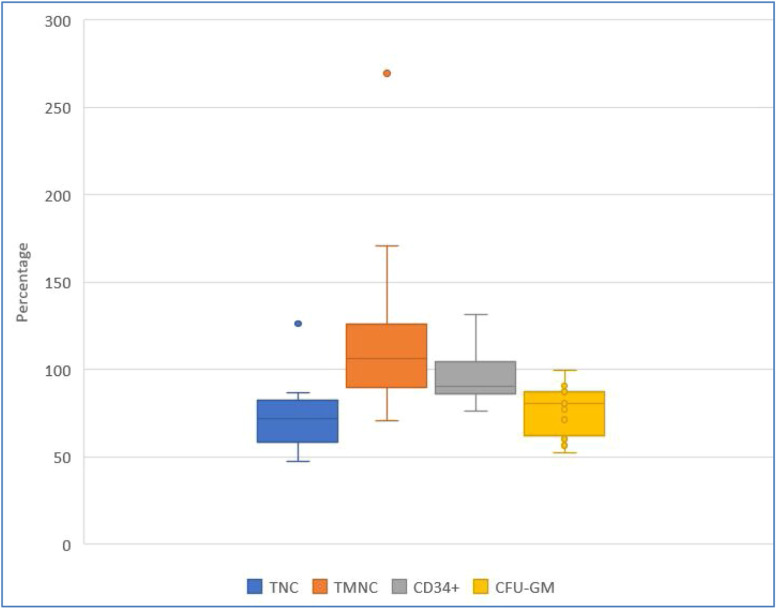
Recovery (%) of key hematopoietic progenitor cell parameters after the freezing/thawing process, comparing data from collection and after freezing/thawing. Blue, orange, and yellow points denote outliers. TNC: total number of viable nucleated cells; TMNC: total number of viable mononuclear cells; CFU-GM: colony forming unit-granulocyte macrophage.

**Figure 3:**
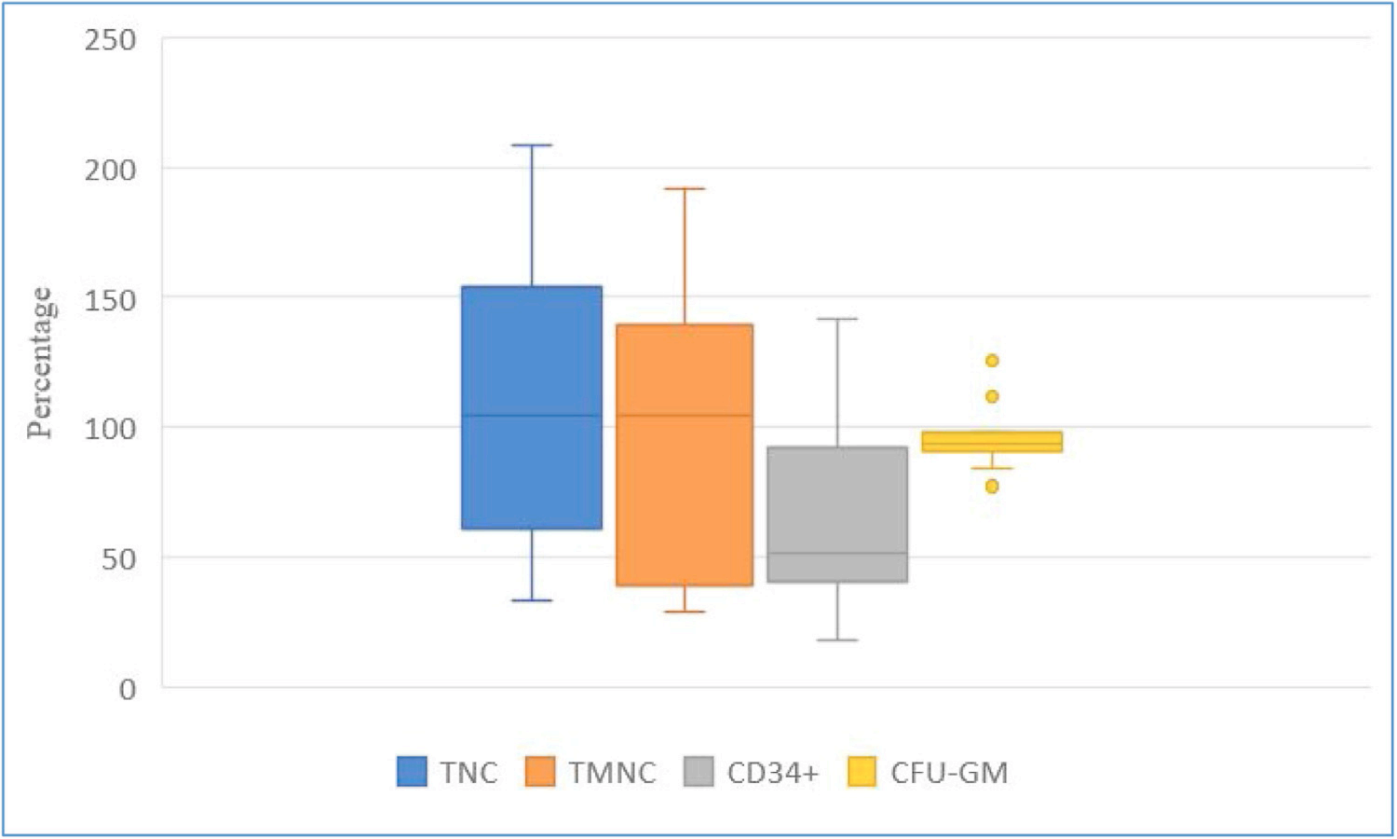
Recovery (%) of key hematopoietic progenitor cell parameters after the dimethyl sulfoxide removal process, comparing data after freezing/thawing process and after dimethyl sulfoxide removal.

TNC: total number of viable nucleated cells; TMNC: total number of viable mononuclear cells; CFU-GM: colony forming unit-granulocyte macrophage

**Figure 4:**
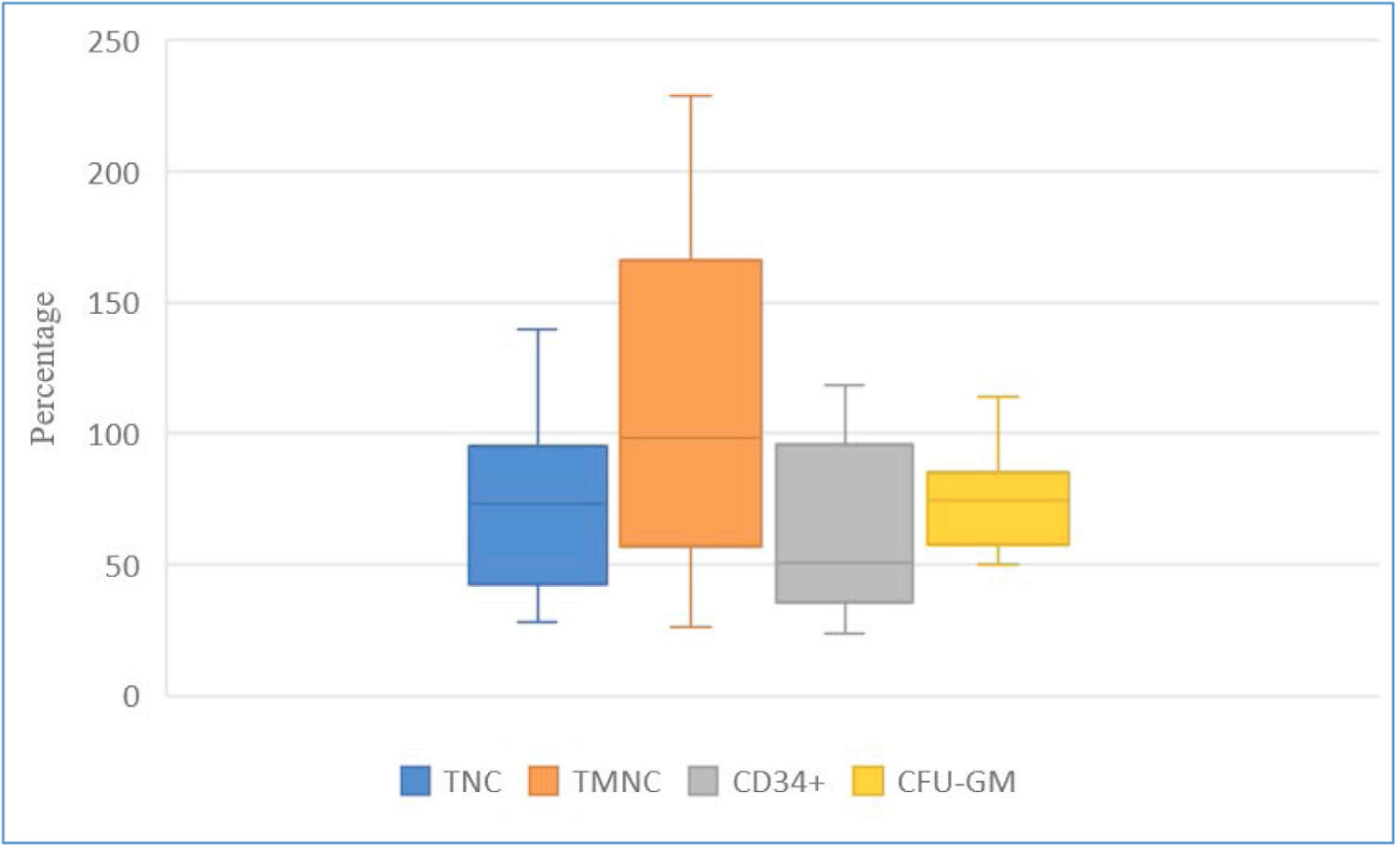
Recovery (%) of key hematopoietic progenitor cell parameters after dimethyl sulfoxide removal, comparing data at collection and after dimethyl sulfoxide removal.

TNC: total number of viable nucleated cells; TMNC: total number of viable mononuclear cells; CFU-GM: colony forming unit-granulocyte macrophage

Figures 2, 3, and 4 show HPC recoveries estimated from the thawed cryobags. Figure 2 shows that after cryopreservation and thawing, the number of all NC was reduced, and CD34^+^ cell potency was also reduced.

Figure 3 shows greater inter-individual differences in cell recovery for TNC, TMNC, and CD34^+^. Recoveries expressed as medians (Q1; Q3) were: TNC 120.85 % (61.16 %; 154.16 %), TMNC 104.53 % (38.98 %;139.27 %), CD34^+^ 51.49 % (40.35 %; 91.82 %) and CFU-GM 93.37 % (90.86 %; 97.59 %) on comparing values ​​obtained after freezing/thawing and after DMSO removal.

Figure 4 shows that DMSO removal decreased TNC, HPC potency and CD34^+^ content. Recoveries, expressed as medians (Q1; Q3), were: TNC 83.98 % (42.62 %; 95.26 %), TMNC 98.71 % (57.00 %; 166.40 %), CD34^+^ 50.69 % (35.90 %; 95.96 %), and CFU-GM 74.80 % (57.92 %; 85.07 %), on comparing values ​​obtained after leukapheresis and after DMSO removal.

### *Results of correlation analysis between CFU-GM and CD34^+^ cells*

**Figure 5:**
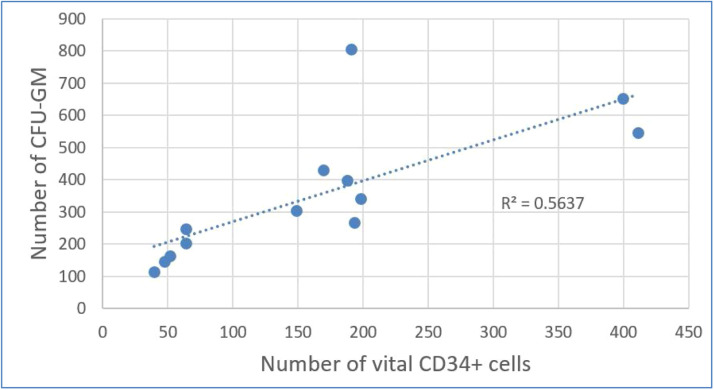
Correlation between post-thaw values of CD34^+^ cell content and colony forming unit-granulocyte macrophage (CFU-GM) content (*r* = 0.751; p-value = 0.003).

**Figure 6:**
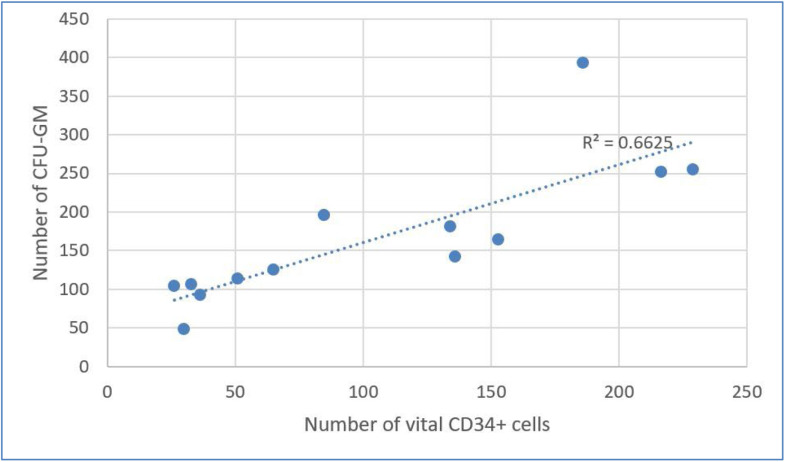
Correlation between the CD34^+^ cell content and colony forming unit-granulocyte macrophage (CFU-GM) content after dimethyl sulfoxide removal (*r* = 0.814; p-value = 0.001).

Table 4 shows considerable decreases in CFU-GM and CD34^+^per kg after DMSO removal. Using Evans Handbook [29], the correlation between post-thaw values of viable CD34^+^ content and CFU-GM content was found to be significant (*r* = 0.751; p-value = 0.003) (Figure 5). A comparable result was found after DMSO removal (*r* = 0.814; p-value = 0.001) (Figure 6).

### *Engraftment of neutrophils and platelets*

In the group of patients with DMSO removal, the engraftment in neutrophils was on average 13.81 ± 2.58 days and of the platelets it was 13.77 ± 2.36 days, which was compliant with the European Society for Blood and Marrow Transplantation (EBMT) Handbook criteria [30]. Engraftment did not exceed the 21-day threshold for any patient.

## Discussion

Glycerol was used as a cryoprotectant in the first autologous hematopoietic cell transplantations performed before the start of the regular hematological transplantation program at University Hospital Hradec Králové, [31,32]. Later, the use of DMSO was introduced, and washing was performed as standard. Since the beginning of 1994, routine washing of DMSO was stopped, and the rule applied abroad was followed, namely that the daily dose of DMSO per kg of the recipient's weight should not exceed 1 g. Nevertheless, certain doubts persisted in the Czech Republic about the clinical use of DMSO, especially with regard to the quality of the product [12]. These doubts were definitively resolved in 1996 when the State Institute for Drug Control fully accepted DMSO for use. This acceptance, however, mandated laboratory testing of DMSO products at individual transplantation centers until a clinically applicable CE-marked product became available in the Czech Republic. If the DMSO daily dose exceeded the recommended limit, the infusions were spread over several days [12].

Currently, there are new trends, the goal of which is either to find other equally effective cryoprotective solutions or to reduce the risk of adverse reactions [15]. In general, the rule of "three Rs" – Replace, Reduce, Remove [33] – is applied. This involves the search for suitable combinations with well-known, but individually less effective, cryoprotectants such as ethylene glycol, hydroxycellulose, sucrose, maltose, trehalose, and also some macromolecules (dextran, polyvinylpyrrolidone, etc.) [13]. Automatic washing systems working in a closed system are available. Compared with classic manual DMSO washing, their advantage is high viability of HPCs and minimal risk of microbial contamination. The disadvantage is the high price of the device [13,17,19,20].

Previous studies at this center with multiple myeloma patients who underwent autologous transplantation demonstrated that DMSO doses per kg administered at transplantation were, in the majority of cases, well below the maximum allowable daily dose [24]. The problem is posed by occasional poorly mobilized patients, with whom it was necessary to split the transplant dose into two after several days.

In accordance with Yang et al. [34], this study demonstrated a correlation between the level of CD34^+^ cells and CFU-GM content. This correlation does not mean, however, that in individual cases such a correlation may not exist as demonstrated by Watts et al. [35] and Morgestern et al. [36]. The results of this study for the CD34^+^ cell freeze/thaw recovery process were higher that the results of Yang et al. [34], who established a median recovery of viable CD34^+^ cells in the freeze/thaw process of 66.4 % (versus 90.18 % in the present study), and CFU-GM of 63.0 % (versus 80.6 % in the present study). This difference can be explained by more efficient cryoprotection based on a combination of DMSO and HES. Mean CD34^+^ cell recovery after manual DMSO removal, as determined by Chen et al. [37] using Trypan blue solution, was 85.4 % which was higher than the present results determined by flow cytometry.

The results of this study were achieved in a relatively small group, as DMSO reduction after thawing was performed only for patients with a known higher risk of arrhythmia (amyloidosis of the heart) or who were at risk of impaired elimination of the DMSO (renal failure caused by amyloidosis of the kidney). In only one case, the removal of DMSO was not planned but was performed in an emergency situation, namely a severe adverse reaction after initiation of an infusion of thawed concentrate.

It was confirmed that the removal of DMSO by washing the cells leads to a significant decrease in the viability of MNCs and the dose of CD34^+^ cells per kg of recipient weight (Table 2) and that the results of the washing process and of the entire process show large individual differences. Decreased viability may be a manifestation of cryopreservation-induced delayed cell death [38]. Nevertheless, in all these patients, a sufficient dose was administered, and delayed engraftments of neutrophils or platelets were not reported. In our practice, we routinely estimate the dose of CD34^+^ cells and CFU-GM from thawed control samples, always comparing the resulting values with the doses determined before cryopreservation.

The results of this study confirm that DMSO washing should be limited to indicated cases only, which is in line with the European Directorate for the Quality of Medicines & HealthCare (EDQM) 2022 recommendation [11]. Another problem is that the result of determining the washed product sterility is known only after administration to the recipient. However, this risk is minimal if the thawed product is handled in purity Grade A clean rooms with Class B background.

We still regard the use of DMSO as safe if the daily dose of 1 g per kg is not exceeded [11].

## Conclusion

DMSO removal should only be performed in indicated cases, as it leads to significant loss of progenitor cells. Despite the fact that data from only 13 patients were analyzed and that the resulting CD34^+^ dose was suboptimal, engraftments were achieved in all cases. The minimal CD34^+^ dose should be 1 × 10^6^ per kg of patient body weight with optimum being 2 × 10^6^ per kg of patient body weight as recommended by EBMT standards. Determination of the CD34^+^ level should be performed simultaneously with determination of CFU-GM to minimize the risk of prolonged engraftment or non-engraftment.

## Financial disclosure statement

This study was supported by MH CZ-DRO (UHHK, 00179906).

## CRediT authorship contribution statement

**Miroslava Jandová:** Writing – original draft, Conceptualization, Data curation. **Pavel Měřička:** Methodology, Writing – review & editing. **Jiří Gregor:** Methodology. **Miriam Lánská:** Visualization. **Aleš Bezrouk:** Formal analysis. **Dana Čížková:** Writing – review & editing. **Jakub Radocha:** Writing – review & editing.

## Conflicts of interest

The author declares no conflicts of interest.
